# Comparison of RNAscope and immunohistochemistry for evaluation of the UPK2 status in urothelial carcinoma tissues

**DOI:** 10.1186/s13000-022-01191-x

**Published:** 2022-01-14

**Authors:** Jiangli Lu, Ming Zhao, Chenyan Wu, Chengbiao Chu, Chris Zhiyi Zhang, Yun Cao

**Affiliations:** 1 Department of Pathology, Sun Yat-sen University Cancer Center, 510060 Guangzhou, P. R. China; 2 State Key Laboratory of Oncology in South China, Collaborative Innovation Center for Cancer Medicine, Sun Yat-sen University, 510060 Guangzhou, P. R. China; 3 Cancer Center, Department of Pathology, Zhejiang Provincial People’s Hospital, Affiliated Peopleʼs Hosipital Hangzhou Medical College, 310014 Hangzhou, Zhejiang, P. R. China; 4 Key Laboratory of Functional Protein Research of Guangdong Higher Education Institutes and MOE Key Laboratory of Tumor Molecular Biology, Institute of Life and Health Engineering, College of Life Science and Technology, Jinan University, 510632 Guangzhou, P.R. China

**Keywords:** UPK2, Urothelial Carcinoma, RNAscope, Immunohistochemistry

## Abstract

**Background:**

UPK2 exhibits excellent specificity for urothelial carcinoma (UC). UPK2 evaluation can be useful in making the correct diagnosis of UC. However, UPK2 detection by immunohistochemistry (IHC) has relatively low sensitivity. This paper aimed to compare the diagnostic sensitivity of RNAscope and IHC for evaluation of the UPK2 status in UC.

**Methods:**

Tissue blocks from 127 conventional bladder UCs, 45 variant bladder UCs, 24 upper tract UCs and 23 metastatic UCs were selected for this study. IHC and RNAscope were used to detect the UPK2 status in UCs. Then, comparisons of the two methods were undertaken.

**Results:**

There was no significant difference between RNAscope and IHC for the evaluation of the UPK2 positivity rate in UC (68.0% vs. 62.6%, *P* = 0.141). Correlation analysis revealed a moderate positive correlation for detection of UPK2: RNAscope vs. IHC (*P* < 0.001, *R* = 0.441). Our results showed a trend toward a higher positive UPK2 rate detected by RNAscope (53.3%) than by IHC (35.6%) in variant bladder UCs. Disappointingly, the *P *value did not indicate a significant difference (*P* = 0.057).

**Conclusions:**

RNAscope for UPK2 appeared to perform similarly to IHC, with a marginally higher positive rate, suggesting it could be used as an alternative or adjunct to UPK2 IHC.

## Introduction

Bladder cancer has become one of the most common carcinomas in China [1]. Urothelial carcinomas (UCs) account for more than 90% of bladder cancers [2]. UC has a marked tendency for divergent differentiation, leading to a variety of histologic variants [3]. It may be difficult to recognize UC variants’ urothelial origin, particularly when variants are present in metastatic sites, since the morphologic features of variants are distinct from those of conventional UC [4]. UPKs comprise a group of four membrane proteins (UPK1a, UPK1b, UPK2, and UPK3a) that are specific differentiation products of urothelial cells [5]. UPK2 is exclusively expressed in the normal urothelium and is undetectable in nonurothelial tissues (skin, prostate, ovary, and liver tissue specimens) [6, 7]. UPK2 is not only expressed specifically in the normal urothelium but also well maintained in UC [8, 9]. UPK2 shows better performance in identifying upper tract UC (UTUC), high-grade UC, and UC variants than UPK3, with more sensitive and equally specific characteristics for UC[10, 11]. Although UPK2 exhibits excellent specificity for UC, UPK2 detection by immunohistochemistry (IHC) has relatively low sensitivity, with 44–80% of cases of conventional invasive UC reported to be positive for UPK2 [4, 11–14]. Therefore, a more reliable and accurate approach is needed. RNAscope (an RNA in situ hybridization assay), a highly sensitive and specific technique, uses a double Z probe strategy that allows simultaneous background suppression and signal amplification to achieve single-molecule visualization while preserving tissue morphology in formalin-fixed, paraffin-embedded (FFPE) tissue sections. [15]. RNAscope is a more sensitive method than IHC for detecting thyroid transcription factor 1 (TTF-1) and Napsin A expression in primary lung adenocarcinomas. RNAscope may be considered for patients with clinically suspected lung adenocarcinoma but who are TTF-1- and Napsin A-negative as detected by IHC[16]. RNAscope improves Glypican3 (GPC3) and glutamine synthetase (GS) specificity and sensitivity by 20-30% in hepatocellular carcinoma. Hepatocellular carcinoma can be diagnosed early via the GPC3 and GS status as detected by RNAscope. [17]. With high specificity, UPK2 detection by IHC plays an important role in the diagnosis of UC. However, this method has relatively low sensitivity. Whether RNAscope can improve the sensitivity for UPK2 detection is unknown at present. This study aimed to evaluate the diagnostic sensitivity of RNAscope compared to IHC for detection of UPK2 in UC.

## Materials and methods

### Samples

Bladder cancer specimens were obtained from the Department of Pathology, Sun Yat-sen University Cancer Center (SYSUCC; Guangzhou, China) between May 2000 and September 2017. We retrieved tissue blocks from 127 conventional bladder UCs (BUCs), 45 variant BUCs, 24 UTUCs, and 23 metastatic UCs. Clinical and pathological information, including age, sex, clinical stage, and histological grade, was collected from medical records. The TNM system (8th AJCC edition) was used to assign the clinical stage.

### Tissue microarray construction

A tissue arrayer (Beecher Instruments, Silver Spring, MD) was used to construct tissue microarrays (TMAs). To obtain new paraffin blocks, two or three representative 1-mm cores were taken from each tissue block. Twenty-one microarrays were constructed from two cores of tumor tissue and one core of normal tissue. Other microarrays were constructed of three cores of tumors. The study protocol was approved by the institutional review board of SYSUCC.

### IHC

Three-micrometer sections were obtained from TMAs and used for immunohistochemical analysis. IHC was performed using an automated staining system (BenchMark ULTRA, Ventana Medical Systems, Inc.) with antibodies against UPK2 (1:100 dilution; BC21, Biocare Medical, Concord, CA) based on the product instructions. Two pathologists evaluated the immunohistochemical slides independently. UPK2 expression was scored as positive if there was cytoplasmic staining present in UC cells.

### RNAscope

RNAscope (Advanced Cell Diagnostics, ACD; Hayward, CA) was performed using probes targeting UPK2 (NM_006760.4) on TMA slides, according to the manufacturer`s protocol. The RNAscope procedure included the following steps. First, the TMA tissue sections were deparaffinized and sequentially subjected to pretreatment 1 (10 min at room temperature), pretreatment 2 (boiling for 20 min), and pretreatment 3 (30 min at 40 °C). Second, slides were hybridized with target probes and incubated in a HybEZ oven (ACD) for 2 h at 40 °C. Third, signals were amplified and generated with an RNAscope 2.0 HD Reagent Kit-BROWN (ACD, Cat. No. 310,035). UPK2 expression was scored as positive if cytoplasmic staining was present in UC cells.

### Statistical analysis

The sensitivity for detection of the UPK2 expression status was evaluated for each method, and comparisons of the two methods were performed using McNemar’s test. Correlation analysis of the two methods was performed using Spearman rank correlation analysis. SPSS 25.0 (version 25.0) was used for all statistical analyses. A two-sided *P* value < 0.05 was considered to be statistically significant.

## Results

### Clinical data

We determined the UPK2 status in 219 samples by both IHC and RNAscope. We analyzed the UPK2 status in 196 patients with primary UC, namely, 162 men (82.4%) and 34 women (17.3%), aged between 27 and 84 years (mean = 62.4). More baseline characteristics of patients with primary UC are summarized in Table 1. We also analyzed the UPK2 status in 23 patients with metastatic UC. More baseline characteristics of patients with metastatic UC are summarized in Table 2.

**Table 1:** Pathologic and clinical features of patients with primary UC

Characteristics	No. (%)
**Patients**	196
**Age** (years)	
Mean (range)	62.4 (27-84)
**Sex**	
Male	162 (82.7)
Female	34 (17.3)
**Stage**	
Tis or Ta	3 (1.5)
T1	5 (2.5)
T2	25 (12.8)
T3	127 (64.8)
T4	36 (18.4)
**Grade**	
Low	9 (4.6)
High	187 (95.4)
**UC type**	
BUC	172 (87.8)
Conventional BUC	127 (64.8)
Variant BUC	45 (23.0)
UTUC	24 (12.2)

Abbreviations: UC, urothelial carcinoma; BUC, bladder urothelial carcinoma; UTUC, upper tract urothelial carcinoma

**Table 2:** Pathologic and clinical features of patients with metastatic UC

Characteristics	No. (%)
**Patients**	23
**Age** (years)	
Mean (range)	60.0 (44-80)
**Sex**	
Male	19 (82.6)
Female	4 (17.4)
**Primary location**	
Bladder	13 (56.5)
Upper tract	10 (43.5)
**Metastatic location**	
Lymph node	20 (87.0)
Others	3 (13.0)
**Grade**	
Low	0 (0.0)
High	23 (100.0)

Abbreviation: UC, urothelial carcinoma

### Correlation of UPK2 status between RNAscope and IHC

**Fig. 1:**
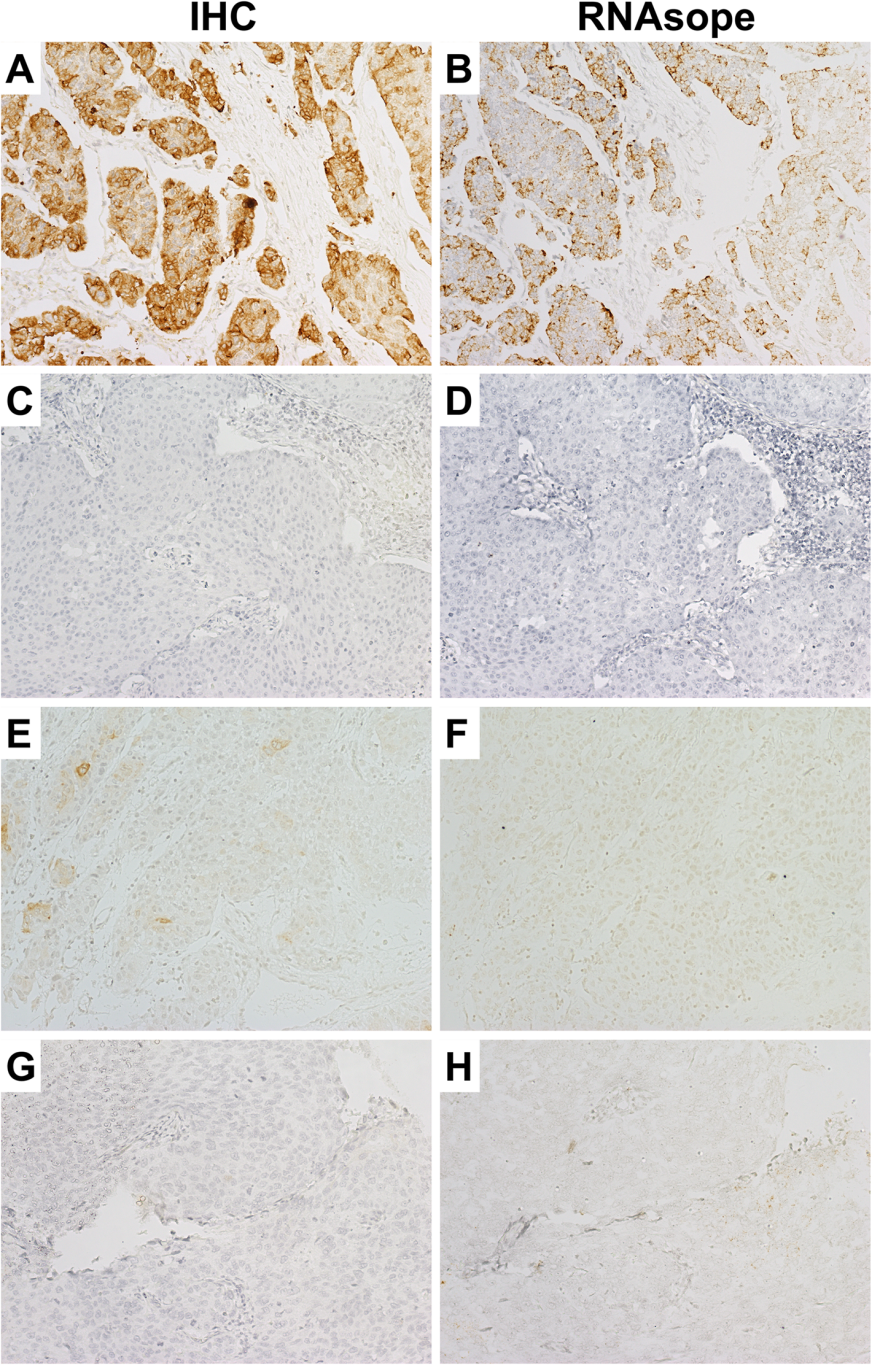
Photomicrographs of urothelial carcinoma in which immunohistochemistry and RNAscope showed similar and different results for the UPK2 status under 200× magnification. **A-B** UPK2 expression was detected to be positive by both IHC and RNAscope. **C-D** UPK2 expression was detected to be negative by both IHC and RNAscope. **E-F** UPK2 expression was evaluated as positive by IHC and negative by RNAscope. **G-H** UPK2 expression was evaluated as negative by IHC and positive by RNAscope

The correlation of the UPK2 status between RNAscope and IHC in UC tissues was evaluated, and the results are shown in Table 3. In these 219 patients, the sensitivity for UPK2 detection by RNAscope was 68.0%, and by IHC was 62.6%. UPK2 expression was detected to be negative by both IHC and RNAscope in 48 patients and was detected to be positive by both IHC and RNAscope in 115 patients (Fig. 1). However, UPK2 expression was evaluated as positive by IHC and negative by RNAscope in 22 patients and negative by IHC and positive by RNAscope in 34 patients. There were no significant differences between RNAscope and IHC in the evaluation of the UPK2 positivity rate in UC (68.0% vs. 62.6%, *P* = 0.141). Correlation analysis revealed a moderate positive correlation in the detection of UPK2: RNAscope vs. IHC (*P* < 0.001, *R* = 0.441). The comparisons of the UPK2 status as determined by IHC and RNAscope in different UC tissues are summarized in Table 4. Although the UPK2 positivity rate detected by RNAscope tended to be higher than that detected by IHC in conventional BUC (72.4% vs. 68.5%, *P* = 0.511) and variant BUC (53.3% vs. 35.6%, *P* = 0.057), the difference was not statistically significant. Furthermore, we found similar UPK2 positivity rates detected by RNAscope and IHC in UTUC (*P* = 1.000) and metastatic UC (*P* = 1.000).

**Table 3:** Crosstabulation of UPK2 status detected by IHC and RNAscope in UC tissues

RNAscope (%)	IHC (%)		Total
	-	+	
-	48 (21.9)	22(10)	70 (32.0)
+	34 (15.5)	115(52.5)	149(68.0)
Total	82 (37.4)	137 (62.6)	219(100.0)

Abbreviations: IHC, immunohistochemistry; UC, urothelial carcinoma

McNemar’s test *P* = 0.141

Spearman rank correlation analysis *P* < 0.001, *R* = 0.441

**Table 4:** Comparison of UPK2 status detected by IHC and RNAscope in different UC tissues

UC Type		Positivity (%)	*P *value
BUC	IHC	103/172 (59.9)	0.092
	RNAscope	116/172 (67.4)	
Conventional BUC	IHC	87/127 (68.5)	0.511
	RNAscope	92/127 (72.4)	
Variant BUC	IHC	16/45 (35.6)	0.057
	RNAscope	24/45 (53.3)	
UTUC	IHC	19/24 (79.2)	1.000
	RNAscope	18/24 (75.0)	
Metastatic UC	IHC	15/23 (65.2)	1.000
	RNAscope	15/23 (65.2)	

Abbreviations: IHC, immunohistochemistry; UC, urothelial carcinoma; BUC, bladder urothelial carcinoma; UTUC, upper tract urothelial carcinoma

## Discussion

Determining the UPK2 status can be useful in making the correct diagnosis of UC; unfortunately, this strategy would fail in many patients in whom UPK2 was detected by IHC, which has unsatisfactory sensitivity in UC, and we sought to determine whether the novel technique RNAscope exhibits improved sensitivity for UPK2 detection in UC. To our knowledge, this is the first study to use RNAscope to detect UPK2 expression in UC and discuss the relationship between IHC and RNAscope for detecting UPK2 expression.

Our results showed that the UPK2 positivity rate detected by IHC was 62.6% in overall UC and 59.9% in BUC. The UPK2 positivity rate in our study was somewhat similar to that in a previous study. UPK2 detection by IHC has different sensitivities, with sensitivities of 44–80% in patients reported by different studies in UC. Tian et al. reported that the UPK2 positivity rate in invasive high-grade UC was 65.6% [14]. Smith et al. noted that UPK2 outperformed UPK3 with greater sensitivity (63% vs. 19%) in bladder neck UC [11]. A UPK2 sensitivity of 77% was shown in a study of 174 invasive BUCs of different stages and grades [12], and a sensitivity of 44% was reported in 105 conventional BUCs [4]. A sensitivity of 80% for UPK2 was reported in a study of 89 patients with muscle-infiltrating UC, although it decreased to 74% when only moderate to intense immunoreactivity was considered [13]. A possible reason for the different results might be that the previous studies included different grades (grades I, II, and III), whereas our study included mainly high-grade UC (95.4%). Different sample sizes may also be responsible for the different sensitivities observed in different studies.

Our results in this study showed that the difference in the detection of UPK2 in UC between RNAscope (68.0%) and IHC (62.6%) was not significant and that there was a moderate positive correlation between RNAscope and IHC (*P* < 0.001, *R* = 0.441) for the detection of UP II. However, RNAscope can improve TTF1 sensitivity by 10.0% and Napsin A sensitivity by 12.5% compared with those of IHC in lung adenocarcinomas [16]. In addition, compared with IHC, RNAscope improved both the specificity and sensitivity for GPC3 and GS by 20–30% in hepatocellular carcinoma [17]. Our attempt to use RNAscope, a novel technique to improve UPK2 sensitivity, was not successful in UC. One reason for this lack of success might be that UPK2 evaluation was performed in TMAs but was performed on whole slides in previous studies. Another reason might be that the UPK2 positivity rate is determined by intratumor molecular characteristics, which results in the recognition of molecularly distinct categories beyond the histopathological classification in UC [18]. Although the molecular subtypes of UC were named differently by various research groups, there was a significant overlap: common basal and luminal molecular subtypes were proposed [19]. In the recently updated bladder TCGA dataset, the basal subtype group contains 35% of the cases, while the combined luminal subtype groups contain 60% [20]. Luminal BUCs are positive for luminal (UPK2, GATA3, and CK20) immunohistochemical markers [21]. Thus, the UPK2 positivity rate is expected to be limited by the proportion of luminal subtype cases. The sensitivity for UPK2 detection by RNAscope was 68% in our study, which was also similar to the percentage of luminal-like subtype samples (56.8%) in our previous study [22]. However, analysis of discordance in 34 samples that were UPK2-negative based on IHC and positive based on RNAscope implied that RNAscope had a potential ability to prevent false negative results for the UPK2 status. The possible reason for the discordance between RNAscope and IHC might be the poor correlation generally reported between mRNA and protein levels. The varied and complicated posttranslational mechanisms involved in translating mRNAs into proteins are not sufficiently explained. In addition, proteins significantly differ in their half-life, and there are technical differences in protein and mRNA detection that result in discordance between protein and mRNA levels [23].

UC, with divergent differentiation, has the potential to differentiate into various types of variants [3]. Despite their unsatisfactory response to therapeutic approaches used for conventional UC, patients with some aggressive variants may benefit from novel therapies [24]. The identification of UC variants has important implications for individualized therapy. Our results showed that the UPK2 positivity rate detected by RNAscope (53.3%) was approximately 20% higher than that detected by IHC (35.6%) in UC variants, and the difference was nearly statistically significant (*P* = 0.057). RNAscope may play a potential role in identifying UC variants. The detection of UPK2 mRNAs was more sensitive than detection of the corresponding proteins for UC variant diagnosis, a difference that may be related to the incomplete translation of biomarker mRNAs into proteins and the delay in dissolution during tissue processing and staining, resulting in partial loss [25]. The reason contributing to the highly specificity and sensitivity of RNAscope for detecting target signals is its double Z probe strategy. The ZZ pair of complementary regions is designed to hybridize to a target RNA. Upon hybridization to the target, the ‘‘tails’’ of each ZZ pair form a sequence complementary to a sequence in the preamplifier probe. Thus, sequential hybridization with the preamplifier, amplifier, and label probes can theoretically amplify each target RNA signal 8000 times [15].

The limitations of the present study include the patient selection and sample size. More patients with variant BUC, UTUC, and metastatic UC should be included. Whole-slide analysis should be performed on samples that are negative for UPK2, because compared to TMA analysis, this method can prevent effects of tumor heterogeneity. Analysis of whole slides may improve the sensitivity of RNAscope for detecting UPK2. Furthermore, as a single-institution study, conclusions from the findings of this study need confirmation in multicenter studies.

In conclusion, RNAscope for UPK2 appeared to perform similarly to IHC, with a marginally higher positive rate, suggesting it could be used as an alternative or adjunct to UPK2 IHC.

## Data Availability

The authenticity of this article has been validated by uploading the key raw data onto the Research Data Deposit public platform (www.researchdata.org.cn), with the RDD approval number RDDB2020000978.
